# Physical Activity under Stress: A Perspective of HAPA and Individual Differences

**DOI:** 10.3390/ijerph182212144

**Published:** 2021-11-19

**Authors:** Song Zhou, Linqian Li, Yan Zhao, Yiheng Cao, Baozhong Peng, Lei Zheng

**Affiliations:** 1 School of Psychology, Fujian Normal University, Fuzhou 350007, China; zhousong@pku.edu.cn (S.Z.); linqian_l@163.com (L.L.); zhaoyanpsy@gmail.com (Y.Z.); 2 School of Physical Education and Sport Science, Fujian Normal University, Fuzhou 350007, China; yihengcao@fjnu.edu.cn; 3 School of Economics and Management, Fuzhou University, Fuzhou 350007, China; pengbaozhongpbz@sina.com

**Keywords:** perceived stress, physical activity, outcome expectancies, future orientation

## Abstract

Aims: Physical activity is an effective way for people to cope with stress. However, people often decrease their physical activity in response to stressors. Therefore, we aimed to understand the relationship between perceived stress and physical activity from an outcome expectancies perspective and investigated the moderating role of future orientation in this relationship. Methods: This study recruited 425 students who completed a three-wave survey at six-week intervals. A moderated mediation model was used to examine the mediating effects of outcome expectancies and the moderating effects of future orientation. Results: The results indicated that outcome expectancies mediated the relationship between perceived stress and physical activity. This relationship was moderated by future orientation. In particular, the mediating effects were significant for people with a high future orientation, but not for those with a low future orientation. Conclusion: Our results demonstrate the adaptive function of future orientation in response to general stress. Importantly, the link between perceived stress and reduced physical activity could be mitigated by encouraging people to focus on future consequences. Future studies should consider developing intervention strategies that help those struggling with stressful contexts.

## 1. Introduction

The rise of health problems among college students has attracted increasing attention in China [1]. One dominant factor that results in physical and psychological problems is perceived stress, which leads to various unhealthy behaviors, such as reduced physical activity [2]. However, participation in physical activity is widely regarded as an effective strategy for coping with stress [3].

According to the Health Action Process Approach (HAPA), outcome expectancies are defined as anticipated health-related consequences resulting from engaging in a particular behavior [4]. Therefore, outcome expectancies form intentions and motivate people to engage in healthy behaviors [5]. Recently, the HAPA model was used to explain behaviors in response to stress [6]. Aligned with this stress management model, this research uses the HAPA model to better understand the association between perceived stress and physical activity. Therefore, we aim to test the mediation effects of outcome expectancies in the relationship between perceived stress and healthy behavior based on the HAPA model.

### 1.1. Literature Review

#### 1.1.1. Perceived Stress and Physical Activity

Stress is a term that is used to describe emotional and physical tension. Numerous studies have shown that stressful experiences may cause adverse effects, including a variety of health consequences [2]. For instance, stress is associated with headaches, muscle pain, depression, and overeating. Physical activity can be an important way to protect the body from the negative effects of stress and to improve mental health outcomes [7]. In fact, exercise has been linked to lower levels of subjective stress [3]; it helps people cope with daily stressors and maintain mental health.

Physical activity is widely regarded as a health-promoting behavior that helps those experiencing stress [3,8]. In fact, the World Health Organization (WHO) recommends that adults between 18 and 64 years of age should be involved in at least 150 min of moderate-intensity aerobic physical activity per week. This reduces symptoms of depression and anxiety and improves health. However, although physical activity is beneficial for both physical and psychological health [2], people do not regard it as their most preferred coping strategy when experiencing stress.

In fact, people tend to adopt unhealthy behaviors, such as reduced physical activity, as an approach to manage their stress [9]. This is because unhealthy behaviors provide temporary relaxation and lead people to overlook the long-term health benefits of physical activity [10]. Cruz et al. [11] proposed that people usually experience negative effects when engaging in physical activity, but experience more positive effects after exercise. As stress causes physical and mental fatigue, people experiencing stress find it more difficult to engage in physical activity [12,13,14]. Consequently, people have reported reduced physical activity when they had to deal with stress [10]. Among student populations, perceived academic stress has also been associated with a lower frequency of physical activity [3,15,16]. Therefore, extra effort is required to overcome fatigue caused by stressors and engage in physical activity [12].

Although people tend to avoid physical activity in response to stress, physical activity is often reported to improve stress reactions and promote quality of life [17] and is widely regarded as a health-promoting approach for mitigating stress [3]. People who engage in more physical activity report lower levels of perceived stress compared to those with less physical activity [18]. Importantly, physical activity helps people to cope with daily stressors and maintain their mental health. For example, a 16-week exercise intervention was found to enhance stress management capacity among firefighters [19]. As physical activity helps people to deal with stress, it is important to identify the psychological variable that motivates physical activity in response to stress.

#### 1.1.2. Outcome Expectancies

HAPA is a psychological theory that describes, explains, and predicts changes in health behavior [8]. It includes three phases: motivation, intention formation, and volition [5,20]. In the motivation phase, outcome expectancies represent the anticipation of the consequences of an upcoming health behavior [8,20]. They play an important role in predicting and forming health-related intentions. For instance, individuals with stronger dieting intentions follow a healthy diet because they experience higher “positive self-image” outcome expectancies than those with weaker intentions.

People may also think about health-related consequences in stressful contexts. Stress is related to impaired physical and psychological functioning [21]. It narrows cognitive bandwidth and directs attention to current needs rather than long-term benefits [22]. However, considering future outcomes may facilitate health-promoting intentions, such as exercise [8,20]. For people with physical disabilities, the lower their negative outcome expectancies are, the more often they participate in physical activities [23]. Studies have shown that regular physical activity can to help mitigate stress [3,24] and prevent age-related diseases [25]. In addition, physical activity stimulates the production of endorphins, which enhance mood, act as natural pain relievers, and reduce the levels of stress hormones such as adrenaline and cortisol [26]. Therefore, thinking about the positive outcomes of physical activity can promote the intention to perform physical activity [20,27].

#### 1.1.3. Future Orientation

Future orientation is a personality construct that refers to stable individual differences in the consideration of the potential future outcomes of one’s current behaviors [5,28,29]. Many studies have shown positive relationships between future orientation and health behaviors [5,30,31,32,33].

Future orientation is regarded as an adaptive factor that facilitates coping with stress. In particular, people with a high future orientation usually report fewer negative outcomes in response to stress compared to those with a low future orientation [34]. This is because future orientation shifts attention to a positive future rather than the current stressor [35], which may lead one to focus on the potential for gain or growth rather than the perceptions of harm and loss [5,28]. Thus, people with a high future orientation tend to show a strong capacity to adjust their behavioral responses after stressful exposure [36]. Therefore, future orientation serves as an adaptive factor that alters the relationship between stress and outcomes related to stressful exposure [34,37].

Future orientation has been found to be related to a wide range of health-promoting behaviors, such as increased physical activity, fruit and vegetable intake, and smoking cessation [5,29,30,32,33]. Accordingly, people who score lower in future orientation are more likely to abandon physical activity to meet their immediate needs and overlook the long-term benefits [32]. In contrast, people with a high future orientation are more likely to engage in physical activity to achieve better physical health and personal happiness [31].

### 1.2. The Present Study

It has become increasingly important to encourage individuals to adopt health-promoting approaches to cope with stress [3]. Previous studies have shown that physical activity can relieve stress and improve long-term health outcomes [3,7,24]. However, people may choose to not perform physical activity in response to stress because it requires extra effort to overcome fatigue caused by a stressor [12]. Therefore, people who encounter stress may perform less physical activity.

H1: Stress is negatively related to physical activity.

When people experience stress, their attention is directed to meeting stress-related needs [22]. They tend to overlook long-term benefits and make short-sighted decisions. However, considering the future outcomes of performing physical activity can motivate people to exercise [8,20]. Therefore, perceived stress may lead to reduced outcome expectancies, resulting in reduced physical activity.

H2: Outcome expectancies mediate the relationship between perceived stress and physical activity.

**Figure 1:**
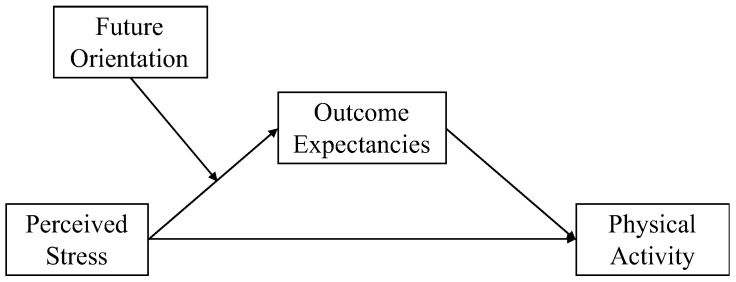
The present study.

Future orientation refers to the extent to which one thinks about the future and considers future outcomes. Having a future orientation helps people to cope with stress and promotes adaptive behavior [34]. People who consider the future consequences of their current behaviors are more likely to engage in behaviors that are good for their future health [38]. Therefore, having a future orientation may serve as a moderator in the association between perceived stress and outcome expectancies (Figure 1).

H3: Having a future orientation moderates the relationship between perceived stress and outcome expectancies.

## 2. Methods

### 2.1. Participants

This study recruited 425 students (225 women and 200 men, mean age = 19.54 ± 1.37) from a university in Fuzhou, China. All participants were undergraduate students.

Participants were asked to complete a three-wave survey after providing online consent, which was approved by the ethics committee of the corresponding author’s university. They completed a perceived stress scale and a physical activity scale, and provided their demographic information at T1. Next, 368 of them finished the outcome expectancies scale and future orientation scale at T2 (6 weeks after T1). Lastly, 293 completed a physical activity scale at T3 (6 weeks after T2). Participants completed all three-wave measures were given extra credit in their course.

### 2.2. Measures

#### 2.2.1. Perceived Stress

Perceived stress was measured by two items adopted from a previous study [39]. The statements were “In the last month, how often have you felt that you were unable to control the important things in your life?” and “In the last month, how often have you felt difficulties were piling up so high that you could not overcome them?”. Participants were instructed to rate on a 5-point scale (0 = never; 4 = very often). A higher mean score indicated a higher level of perceived stress. The Cronbach’s alpha was 0.76 in this study.

#### 2.2.2. Physical Activity

Physical activity was measured by two items adopted from Schwarzer’s research [40]. They were asked to rate on a 5-point scale (0 = none; 1 = 1–2 times; 2 = 3–4 times; 3 = 5–6 times; and 4 = 7 times or above). One of the items was “In the last month, how often did you engage in *physical activities?”. Participants responded on a 5-point scale (0 = none; 1 = rarely; 2 = sometimes; 3 = often; 4 = very often). People who reported a higher mean score indicated that they engaged in more physical activity. The Cronbach’s alpha was 0.85 for Time 1 and 0.88 for Time 3 in this study.

#### 2.2.3. Outcome Expectancies

Outcome expectancies were measured by three items adopted from a prior research study [40]. The statements include “If I engage in physical activities, I can prevent diseases”, “If I engage in physical activities, my friends will see that I am a health-conscious person”, and “If I engage in physical activities, I feel attractive”. The items were rated on a 5-point scale (1 = extremely disagree; 5 = extremely agree). The Cronbach’s alpha was 0.92 in this sample. Those who scored higher were more inclined to think about the consequences of the exercise.

#### 2.2.4. Future Orientation

Future orientation was measured by the consideration of future consequences scale [41]. Five items measured future orientation and seven items measured present orientation. In this study, a future orientation subscale was used (e.g., “I consider how things might be in the future and try to influence those things with my day-to-day behavior”). The Cronbach’s alpha for this study was 0.82. People with high scores were more likely to adopt a future-thinking perspective.

## 3. Results

### 3.1. Attrition Analysis

The results showed that there were no significant mean differences between the follow-up sample and the drop-out sample (perceived stress: t[423] = 1.63, *p* = 0.104, Cohen’s d = 0.16; physical activity: t[423] = 1.64, *p* = 0.103, Cohen’s d = 0.16; outcome expectancies: t[366] = 0.50, *p* = 0.621, Cohen’s d = 0.05).

### 3.2. Mediating Effect of Outcome Expectancies

To examine the relationship between perceived stress and physical activity, we conducted a regression model with physical activity (T3) as the dependent variable and baseline physical activities and demographic variables (T1) as control variables. The results showed that there were significant effects for women (β = −0.29, s.e. = 0.12, *p* = 0.012), education (β = 0.50, s.e. = 0.12, *p* < 0.001), and baseline physical activity (β = 0.41, s.e. = 0.06, *p* < 0.001), but no significant effects for age (β = 0.01, s.e. = 0.04, *p* = 0.776). Subsequently, we included perceived stress in the model. As H1 hypothesized, the results showed that perceived stress was negatively associated with physical activity (β = −0.22, s.e. = 0.06, *p* < 0.001, R^2^ = 0.04).

**Figure 2:**
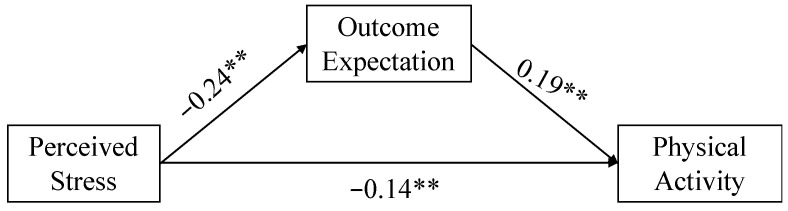
The mediating effect of outcome expectancies. ** *p* < 0.01.

Next, we conducted a mediation model using the lavaan package. As shown in Figure 2, outcome expectancies were positively related to physical activity (β = 0.19, s.e. = 0.06, *p* = 0.001). Perceived stress was negatively associated with both outcome expectancies (β = −0.24, s.e. = 0.08, *p* = 0.002) and physical activity (β = −0.14, s.e. = 0.07, *p* = 0.043). The indirect effect was estimated by using the bootstrap approach with 5000 resamplings. As H2, the results indicated a significant mediating effect of outcome expectancies (T2) in the relationship between perceived stress (T1) and physical activity (T3) (indirect effect coefficient = −0.05, s.e. = 0.02, 95%CI [−0.11, −0.01]).

### 3.3. Moderating Effect of Future Orientation

**Figure 3:**
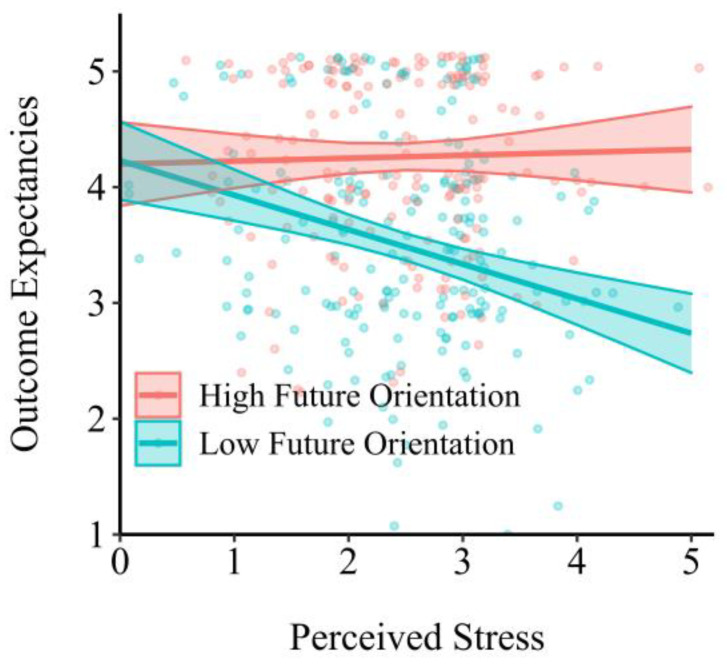
The moderating effect of future orientation.

To examine the moderating effect of future orientation, we constructed a moderated mediation model. Our results showed that future orientation moderated the relationship between perceived stress and outcome expectancies (β = 0.24, s.e. = 0.07, *p* < 0.001). The simple slope analysis showed that the association between stress and outcome expectancies was significant for people with a low future orientation (β = −0.23, s.e. = 0.08, *p* = 0.002) but not for people with a high future orientation (β = −0.08, s.e. = 0.07, *p* = 0.249; Figure 3).

Subsequently, we estimated the moderated mediation effect with 20,000 simulations using the Monte Carlo approach. As per H3, our results showed that future orientation moderated the mediation effect of outcome expectancies on the relationship between perceived stress and physical activities (moderated mediation effect coefficient = 0.04, s.e. = 0.02, 95%CI [0.01, 0.07]). For people with a low future orientation, perceived stress at T1 predicted outcome expectancies at T2 (β = −0.36, s.e. = 0.10, *p* < 0.001), which subsequently predicted physical activity at T3 (β = 0.23, s.e. = 0.08, *p* = 0.003), suggesting a mediating effect of outcome expectancies (indirect effect coefficient = −0.08, s.e. = 0.04, 95%CI [−0.16, −0.001]). However, for people with a high future orientation, this study did not detect a significant mediating effect (indirect effect coefficient = −0.01, s.e. = 0.02, 95%CI [−0.05, 0.02]).

## 4. Discussion

This study found that perceived stress is negatively related to physical activity, which is consistent with the results of previous studies [11]. One intriguing finding is that outcome expectancies serve as a mediator in the relationship between stress and physical activity. This relationship varies under the conditions of future orientation. Specifically, people with a high future orientation perceived that stress negatively affected their outcome expectancies, which subsequently positively predicted their physical activity. This is in line with the findings of previous studies, which posit that when people consider the future consequences of their choices, they tend to engage in health-promoting behaviors [5].

### 4.1. Outcome Expectancies Explain Associations between Perceived Stress and Physical Activity

Our results demonstrated a negative relationship between stress and physical activity. This is in line with previous studies that demonstrate the relationship between academic stress and a reduced frequency of physical activity [9,11]. When people are faced with stress, they are more likely to adopt unhealthy behaviors to cope with the tension caused by stress, such as exercising less and eating more unhealthy foods. This study explains why physical activity can elicit feelings of freedom and relaxation and thus reduce anxiety and stress. Physical activity shifts one’s attention from the work environment, for example, and promotes a sense of recovery from a stressful state [3,42]. In addition, the stress caused by a heavy workload of exams and lectures often leads to reduced exercise to meet academic demands [3,15,16].

In this study, outcome expectancies served as a mediator in the relationship between stress and physical activity. Several studies have attempted to understand the effects of stress on reduced health behaviors such as physical activity [9,11]. In the HAPA model, outcome expectancies underlie the motivation system of behaviors, which reminds individuals to consider health-related consequences when confronted with a stressful situation. The consideration of future results may assist in the adoption of health-promoting activities, such as exercise. The lower their negative result expectations are, the more often persons with physical limitations engage in physical activities. Additionally, as physical exercise aids in stress reduction and promotes long-term health results [7], contemplating the benefits of physical exercise when experiencing stress may help increase the intention to engage in physical activity. Our results suggest that stress is related to lower outcome expectancies, resulting in reduced physical activity. Outcome expectancies promote stress management [6]. This study provides empirical evidence about the role of outcome expectancies in the relationship between stress and physical activity. Furthermore, stress can result in limited cognitive resources [43], making people pay attention to current pressures and overlook long-term costs. Therefore, people with high levels of perceived stress are less likely to consider the future consequences of the lack of physical activity, which in turn further reduces present physical activity.

### 4.2. Future Orientation Alters Effects of Perceived Stress on Outcome Expectancies

Future orientation was found to buffer individuals from the effects of stress and promote outcome expectancies in this study. Individuals with a high future orientation tend to prepare for future events in advance [28]. This personality trait seems to motivate people to engage in pursuing “self-actualized” goals, as proposed by Abraham Maslow [44]. Generally, self-actualized individuals have a sense of purpose and perform routine tasks that contribute to that objective. When individuals consider the future consequences of exercise, their long-term goals motivate them to engage in activities to achieve self-actualization. Therefore, future-oriented people tend to engage in more health-promoting behaviors [5].

Importantly, the link between perceived stress and reduced physical activity could be mitigated by shifting attention to future consequences. The results provide evidence for the adaptive function of future orientation in a general stressful context. Individuals who focus on the future are more likely to engage in physical activities. As physical activity helps one to cope with stress [3], future orientation helps people reduce the potential adverse effects of stress, leading to adaptive behaviors [37]. Future orientation as an adaptive factor can lead individuals to shift their attention to a positive future, which prompts them to transfer their concerns about the present to physical activity. Individuals with a strong future orientation often report fewer negative consequences in reaction to stress than those with a low future orientation [34], which is consistent with our findings. This is because future orientation directs attention away from the present stressor and towards a more positive future [35], which may encourage one to concentrate on the possibility of gain or progress rather than on perceived injury or loss. Thus, individuals with a strong future orientation often have an exceptional ability to alter their behavioral reactions after stressful exposure. Therefore, future orientation acts as a moderating element in the link between stress and the effects associated with stressful exposure. Specifically, having a low future orientation buffers individuals from the negative effects of perceived stress on physical activity. However, people with a high future orientation, regardless of their level of perceived stress, tend to consider the outcome of the lack of physical movement, which further affects their intention to exercise. Therefore, people with a high future orientation are more willing to think about the positive outcomes of exercising, which motivates them to implement healthy behaviors and helps them to alleviate the detrimental effects of stress.

### 4.3. Implications

In this study, the link between perceived stress and reduced physical activity was mitigated by shifting attention to future consequences. In line with this, a strategy that promotes future orientation has been found to help individuals cope with the psychological stress caused during the COVID-19 pandemic [45]. Our results also suggest that future-oriented interventions can promote physical activity by altering outcome expectancies in stressful contexts. In addition, our findings suggest that stress management interventions should consider individual differences, as the relationship between stress and physical activity varies under the influence of future orientation. More attention should be paid to people with a low future orientation, since they are more likely to reduce or avoid engaging in physical activity in response to stress.

### 4.4. Limitations

This study has several limitations. First, this study conducted a three-wave longitudinal survey solely based on self-reported measurements, and causal inference could not be determined. As long questionnaires often lead to negative moods, fatigue, and unwillingness to continue, we adopted two or three items in this study. Although the reliability of the scales was acceptable, future studies are needed to examine our results via widely used scales. Second, this study solely investigated the effects of general stress on physical activity. Other stressors, such as short-term stress, may have different effects on healthy behaviors. Accordingly, the psychological mechanisms underlying these relationships may differ across various stressors. Third, the adjustment effect of exercise intensity was not considered in our study. There may be a reverse U-shaped relationship between exercise intensity and outcomes related to physical activity. Future research could aim to further explore the role of this variable in the mechanisms underlying this relationship.

## 5. Conclusions

This study suggested the adaptive function of future orientation in response to general stress among university students. Our results highlighted the mediation role of outcome expectancies in the link between perceived stress and reduced physical activity. Importantly, such relationship could be mitigated by encouraging people to focus on future consequences.

## Data Availability

The data presented in this study are available on request from the corresponding author.
